# Mask-Ematics: Modeling the Effects of Masks in COVID-19 Transmission in High-Risk Environments

**DOI:** 10.3390/epidemiologia2020016

**Published:** 2021-05-31

**Authors:** Anthony Morciglio, Bin Zhang, Gerardo Chowell, James M. Hyman, Yi Jiang

**Affiliations:** 1 Department of Mathematics and Statistics, Georgia State University, Atlanta, GA 30303, USA; amorciglio1@student.gsu.edu (A.M.); bzhang17@student.gsu.edu (B.Z.); 2 Department of Public Health, Georgia State University, Atlanta, GA 30303, USA; gchowell@gsu.edu; 3 Department of Mathematics, Tulane University, New Orleans, LA 70118, USA; mhyman@tulane.edu

**Keywords:** COVID-19, masks, mathematical model, ordinary differential equations, reproductive number, compartment model, Diamond Princess

## Abstract

The COVID-19 pandemic has placed an unprecedented burden on public health and strained the worldwide economy. The rapid spread of COVID-19 has been predominantly driven by aerosol transmission, and scientific research supports the use of face masks to reduce transmission. However, a systematic and quantitative understanding of how face masks reduce disease transmission is still lacking. We used epidemic data from the Diamond Princess cruise ship to calibrate a transmission model in a high-risk setting and derive the reproductive number for the model. We explain how the terms in the reproductive number reflect the contributions of the different infectious states to the spread of the infection. We used that model to compare the infection spread within a homogeneously mixed population for different types of masks, the timing of mask policy, and compliance of wearing masks. Our results suggest substantial reductions in epidemic size and mortality rate provided by at least 75% of people wearing masks (robust for different mask types). We also evaluated the timing of the mask implementation. We illustrate how ample compliance with moderate-quality masks at the start of an epidemic attained similar mortality reductions to less compliance and the use of high-quality masks after the epidemic took off. We observed that a critical mass of 84% of the population wearing masks can completely stop the spread of the disease. These results highlight the significance of a large fraction of the population needing to wear face masks to effectively reduce the spread of the epidemic. The simulations show that early implementation of mask policy using moderate-quality masks is more effective than a later implementation with high-quality masks. These findings may inform public health mask-use policies for an infectious respiratory disease outbreak (such as one of COVID-19) in high-risk settings.

## 1. Introduction

COVID-19, the respiratory disease caused by the SARS-CoV-2 virus, has caused an unprecedented burden on the global economy, health, and general well-being [1]. Face masks, social distancing, hand washing, and frequent testing are the most effective ways to slow the spread of SARS-CoV-2 until an effective vaccine is widely available [2]. Policymakers and the public need urgent guidance on the use of masks by the general population as a tool to impede COVID-19 transmission. Recent studies validated that face masks effectively mitigate the spread of COVID-19 [3]. Even so, the adoption by some parts of the world, especially the United States, followed in staggered and hesitant steps. The resistance to wearing a mask is rooted in complex cultural and political considerations, including an initial global shortage of N95 respirators and surgical masks in hospitals [4].

We now understand that the SARS-CoV-2 virus replicates in the upper respiratory tract [5,6], and that viral transmission occurs predominantly through respiratory droplets [7]. Droplets emitted from singing, coughing, sneezing, talking, and even breathing [8] have distinct sizes: larger ones (>10 μm) can land on a person’s eyes, nose, or mouth in near proximity, or quickly fall into surfaces due to gravity; smaller ones (0.2 μm–10 μm, also termed aerosols) can linger in the air for hours. Even with the lockdown interventions, the virus can spread through poorly ventilated buildings [7], as shown in the outbreak on the cruise ship Diamond Princess [9]. Mounting evidence shows presymptomatic and asymptomatic individuals contribute significantly to the spread of COVID-19 [10].

At the beginning of the COVID-19 pandemic, the support for mask-mitigated reduction in viral infection was controversial. Although there have been limited clinical trials, many experimental studies have suggested that masks can reduce viral transmission by blocking a susceptible person’s exposure to respiratory droplets and reducing the spread of viral particles from infected people [11,12,13]. In a study of two hairstylists infected with COVID-19, they did not infect any of their 139 clients or six coworkers who also wore masks [14]. This study is far from definitive, but it supports the effectiveness of consistent face coverings in reducing the spread of SARS-CoV-2. One controlled trial of mask use found masks have a protective efficacy for influenza of 80% [15]. More studies have demonstrated the efficacy of face masks in blocking both particles transmitted by the wearer [16] and particles received by the wearer [17]. Reducing the infectious dose can also significantly reduce the severity of the symptoms resulting from the infection [18].

Based on the evidence available, it appears that wearing masks in public can reduce the spread of COVID-19, although the magnitude of reduction in SARS-CoV-2 transmission is unclear [19]. A few mathematical models have been developed to determine the effectiveness of wearing face masks in reducing the early spread of the infection [20,21,22,23,24,25]. One study used a differential equation model that divided the population into susceptible, exposed, infected, asymptomatic, and recovered (SEIAR) groups and considered mask wearing in relation to cumulative mortality and hospitalization [21]. Their results suggest that even widespread usage of moderate-quality masks is sufficient to reduce hospitalization and deaths. Another study used a branching process to evaluate the discrete timing of mask implementation, and statistical analysis of the basic reproductive number [20].

All models showed that increasing the public’s mask use could significantly reduce the rate of COVID-19 spread, yet they were limited by not considering the timing of mask policy. Maximum effectiveness was attained when everyone wore a mask in the model, and minimal effectiveness resulted when less than half of the population wore masks. A state-level transmission model predicted that hundreds of thousands of lives could be saved by the end of February of 2021 in the United States if universal mask use could be achieved [24].

We used a SEIAR model to study the efficacy of masks as a function of the fraction of the population wearing face masks and the timing of mask implementation in a high-risk setting. We derived an analytical expression for the basic and effective reproductive numbers that elaborates the contribution of each infected sub-population.

We chose to apply the model to the Diamond Princess cruise ship outbreak because it was a dense population in an encapsulated environment, representing a high-risk setting, and because the time course of the outbreak was carefully documented [9,26]. Since the cruise ship passengers did not wear masks, the Diamond Princess serves as an experimental control for mask-mediated mitigation of infection. Our model shows that a certain minimum fraction of people need to wear masks to effectively slow the spread of the infection. This threshold fraction depends on the types of masks. Although a large population wearing N95 masks shows the most significant reduction in infection-induced mortality, moderate-quality masks (e.g., cloth masks) provide similar benefits when worn early and by a larger fraction of the population.

## 2. Materials and Methods

**Figure 1:**
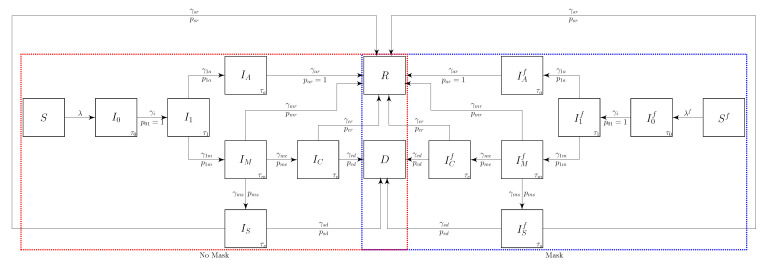
Block flow diagram for the COVID-19 transmission epidemic model. The superscript *f* corresponds to compartments of individuals wearing face masks, and the subscripts for the infected compartments identify their stage in the epidemic cycle. The susceptible and infected populations are divided into non-mask wearers for the susceptible, infected (not infectious), presymptomatic infectious, asymptomatic infectious, mildly symptomatic, severe illness, critical illness, and recovered compartments ($S,I_{0},I_{1},I_{a},I_{m},I_{s},I_{c},R$). The susceptible (*S* or $S^{f}$) is infected at the rate (force of infection) of λ, or $r^{s}\lambda^{f}$ per day. The transition probability, $P_{jk}$, is the propensity that a person goes from compartment *j* to *k* at the rate γjk.

We define the stratified COVID-19 transmission model with masks (Figure 1) by dividing the population into susceptible, *S*, infected, $I_{*}$, and recovered, *R*. The infected group is further divided based on the disease progression. The groups wherein people wear masks are indicated by the superscript *f*, and the infected groups are indicated by the subscript.

Individuals in the susceptible compartments are infected with a force of infection, λ or $r^{s}\lambda^{f}$, that depends on the effectiveness of the face masks. Once infected, they progress into an early-infected, but not infectious stage, $I_{0}$. After an average of $\tau_{0}$ days, they progress into a presymptomatic infectious stage, $I_{1}$, with rate $\gamma_{01}$. We assume that the wearing of face mask s does not change when a person progresses to a new compartment.

An individual stays in the presymptomatic stage for an average of $\tau_{1}$ days, after which a fraction, $P_{1a}$, progresses to the asymptomatic spreader stage, $I_{a}$, at the rate $\gamma_{1a}$. The remaining fraction $P_{1s}=1-P_{1A}$ progresses and develop mild symptoms at the rate $\gamma_{1m}$ to enter stage, $I_{m}$. After an average of $\tau_{a}$ days, the symptomatic individuals recover and enter the recovered state *R* at the rate of $\gamma_{ar}$. After an average of $\tau_{m}$ days, individuals with mild symptoms either severe symptoms, $I_{s}$, with probability the $P_{ms}$ at the rate $\gamma_{ms}$; develop critically severe symptomatic, $I_{c}$, with probability the $P_{mc}$ at the rate $\gamma_{mc}$; or recover (*R*) with probability the $P_{mr}=1-P_{mc}-P_{mc}$ at the rate $\gamma_{mr}$.

We also include a deceased compartment, *D*, in the model and distinguish between symptomatic severe, $I_{s}$, and critical, $I_{c}$ based on their mortality. The branching probabilities $P_{sd}$ and $P_{cd}$ fractions reflect the different mortality rates $\gamma_{sd}$ and $\gamma_{cd}$. This flexibility allowed the model to fit both the Diamond Princess infection and mortality data simultaneously.

The forces *of* infection in block diagram (Figure 1) represent the rates, λ and $r^{s}\lambda^{f}$, that the susceptible population is being infected. The forces *from* infection, $\alpha_{j}$, represent the rate at which an infectious person in $I_{j}$ is infecting others. The force from infection viewpoint provides better insight into understanding the relative importance of infectious compartments in an epidemic.

### 2.1. Differential Equation Model

We formulate the system of ordinary differential equations corresponding to the block diagram in Figure 1 from both view points as

$$\begin{array}{cc} \frac{dS}{dt}= & -\lambda \;S\\ = & -\left(\alpha_{1}I_{1}+\alpha_{a}I_{a}+\alpha_{m}I_{m}+\alpha_{s}I_{s}+\alpha_{c}I_{c}\right)\\ \; & -r^{i}\left(\alpha_{1}I_{1}^{f}+\alpha_{a}I_{a}^{f}+\alpha_{m}I_{m}^{f}+\alpha_{s}I_{s}^{f}+\alpha_{c}I_{c}^{f}\right)\\ \frac{dI_{0}}{dt}= & \lambda S-\gamma_{01}I_{0}\\ = & \left(\alpha_{1}I_{1}+\alpha_{a}I_{a}+\alpha_{m}I_{m}+\alpha_{s}I_{s}+\alpha_{c}I_{c}\right)\\ \; & +r^{i}\left(\alpha_{1}I_{1}^{f}+\alpha_{a}I_{a}^{f}+\alpha_{m}I_{m}^{f}+\alpha_{s}I_{s}^{f}+\alpha_{c}I_{c}^{f}]\right)-\gamma_{01}I_{0}\\ \frac{dI_{1}}{dt}= & \gamma_{01}I_{0}-\left(\gamma_{1a}+\gamma_{1m}\right)I_{1}\\ \frac{dI_{a}}{dt}= & \gamma_{1a}I_{1}-\gamma_{ar}I_{a}\\ \frac{dI_{m}}{dt}= & \gamma_{1m}I_{1}-\left(\gamma_{mr}+\gamma_{ms}+\gamma_{md}\right)I_{m}\\ \frac{dI_{s}}{dt}= & \gamma_{ms}I_{m}-\left(\gamma_{sr}+\gamma_{sd}\right)I_{s}\\ \frac{dI_{c}}{dt}= & \gamma_{mc}I_{c}-\left(\gamma_{cd}+\gamma_{cr}\right)I_{c}\\ \frac{dS^{f}}{dt}= & -r^{s}\lambda^{f}\;S\\ = & -r^{s}\left(\alpha_{1}^{f}I_{1}+\alpha_{a}^{f}I_{a}+\alpha_{m}^{f}I_{m}+\alpha_{s}^{f}I_{s}+\alpha_{c}^{f}I_{c}\right)\\ \; & -r^{s}r^{i}\left(\alpha_{1}^{f}I_{1}^{f}+\alpha_{a}^{f}I_{a}^{f}+\alpha_{m}^{f}I_{m}^{f}+\alpha_{s}^{f}I_{s}^{f}+\alpha_{c}^{f}I_{c}^{f}\right)\\ \frac{dI_{0}^{f}}{dt}= & r^{s}\lambda S^{f}-\gamma_{01}I_{0}^{f}\\ = & r^{s}\left(\alpha_{1}^{f}I_{1}+\alpha_{a}^{f}I_{a}+\alpha_{m}^{f}I_{m}+\alpha_{s}^{f}I_{s}+\alpha_{c}^{f}I_{c}\right)\\ \; & +r^{s}r^{i}\left(\alpha_{1}^{f}I_{1}^{f}+\alpha_{a}^{f}I_{a}^{f}+\alpha_{m}^{f}I_{m}^{f}+\alpha_{s}^{f}I_{s}^{f}+\alpha_{c}^{f}I_{c}^{f}\right)-\gamma_{01}I_{0}^{f}\\ \frac{dI_{1}^{f}}{dt}= & \gamma_{01}I_{0}^{f}-\left(\gamma_{1a}+\gamma_{1m}\right)I_{1}^{f}\\ \frac{dI_{a}^{f}}{dt}= & \gamma_{1a}I_{1}^{f}-\gamma_{ar}I_{a}^{f}\\ \frac{dI_{m}^{f}}{dt}= & \gamma_{1m}I_{1}^{f}-\left(\gamma_{mr}+\gamma_{ms}+\gamma_{md}\right)I_{m}^{f}\\ \frac{dI_{s}^{f}}{dt}= & \gamma_{ms}I_{m}^{f}-\left(\gamma_{sr}+\gamma_{sd}\right)I_{s}^{f}\\ \frac{dI_{c}^{f}}{dt}= & \gamma_{mc}I_{m}^{f}-\left(\gamma_{cd}+\gamma_{cr}\right)I_{c}^{f}\\ \frac{dR}{dt}= & \gamma_{ar}\left(I_{a}+I_{a}^{f}\right)+\gamma_{mr}\left(I_{m}+I_{m}^{f}\right)+\gamma_{sr}\left(I_{s}+I_{s}^{f}\right)+\gamma_{cr}\left(I_{c}+I_{c}^{f}\right)\\ \frac{dD}{dt}= & \gamma_{sd}\left(I_{s}+I_{s}^{f}\right)+\gamma_{cd}\left(I_{c}+I_{c}^{f}\right)\;. \end{array}$$

#### 2.1.1. Force of Infection

The force of infection, λ, on the non-mask wearing susceptible population, *S* is the rate at which the population in *S* is being infected. This rate can be expressed as the sum of the forces of infection from each of the infectious compartments:

$$\lambda =\lambda_{1}+\lambda_{1}^{f}+\lambda_{a}+\lambda_{a}^{f}+\lambda_{m}+\lambda_{m}^{f}+\lambda_{s}+\lambda_{s}^{f}+\lambda_{c}+\lambda_{c}^{f}\;.$$

The force of infection coming from a person in the non-mask wearing *k* infectious compartment is

$$\begin{array}{ccc} \lambda_{j} & = & c_{S}\times \beta_{j}\times \;P_{j}\\ & = & \left(\begin{array}{c} \mathrm{Number}\;\mathrm{of}\\ \mathrm{daily}\\ \mathrm{susceptible}\\ \mathrm{contacts} \end{array}\right)\times \left(\begin{array}{c} \mathrm{Probability}\;\mathrm{of}\\ \mathrm{transmission}\\ \mathrm{per}\;\mathrm{contact}\;\mathrm{with}\\ \mathrm{susceptible}\;\mathrm{in}\;I_{j} \end{array}\right)\times \left(\begin{array}{c} \mathrm{Probability}\\ \mathrm{that}\;a\\ \mathrm{random}\;\mathrm{contact}\\ \mathrm{is}\;\mathrm{in}\;I_{j} \end{array}\right)\;. \end{array}$$

Note that we have used the notation $c_{S}$ for the number of contacts for the susceptible population to differentiate it from the number of contacts, $c_{s}$, for someone in $I_{s}$. A contact is any activity where an infectious person can infect a susceptible person. The infectious people in $I_{j}$ have $c_{j}I_{j}$ total contacts, and we assume that the mask wearing does not affect the contact rates.

The transmissibility, $\beta_{j}$, is the probability of a non-mask-wearing susceptible person infected by a single contact with a non-mask wearing infectious person in $I_{j}$. The infectiousness of infected face-masked individuals decreases since the mask blocks a proportion of the aerosol particles [20]. We assume that the transmissibility is reduced by the factor $r^{i}$ for an infectious person wearing a mask. Similarly, the transmissibility of the infection to susceptible people wearing a mask is reduced by $r^{s}$.

We assume the population is mixing randomly. The probability that a random contact is with someone in $I_{j}$ is the ratio of the number of contacts, $c_{j}$, that the people in $I_{j}$ have per day, $c_{j}I_{j}$, divided by the total number of contacts in the entire population,

$$C_{tot}\left(t\right)=c_{S}S+c_{S}S^{f}+\sum_{j}c_{j}I_{j}+\sum_{j}c_{j}I_{j}^{f}+c_{R}R\;.$$

Here, the sums are over all of the compartments that have contacts. The proportion of the random contacts that are with someone in $I_{j}$ is $P_{j}=c_{j}I_{j}/C_{tot}\left(t\right)$. We assume that the number of contacts is independent of wearing a facial mask.

If the susceptible person has contact with a face-mask-wearing infected person, the transmissible is reduced by $r^{i}$. The resulting force of infection from infectious mask-wearing individuals is $\lambda_{j}^{f}=c_{S}\left(r^{i}\beta_{j}\right)P_{j}^{f}$, where $P_{j}^{f}=c_{j}I_{j}^{f}/C_{tot}\left(t\right)$ is the fraction of the contacts with $I_{j}^{f}$. The force of infection on the mask wearing susceptible population, $S^{f}$ is further reduced by $r^{s}$ and can be expressed as $r^{s}\lambda^{f}$.

#### 2.1.2. Force from Infection

Evaluating the model from the infectious population viewpoint can help clarify the roles of the different infectious stages in spreading the epidemic and simplifies the analysis for the effective reproductive number. The force from infection, $\alpha_{j}$, is the rate at which an infectious person in compartment *j* is infecting susceptible people can be defined for each infectious compartment as

$$\begin{array}{ccc} \alpha_{j} & = & c_{j}\times \beta_{j}\times P_{S}\\ & = & \left(\begin{array}{c} \mathrm{Number}\;\mathrm{of}\\ \mathrm{daily}\\ \mathrm{contacts}\;\mathrm{an}\\ \mathrm{individual}\;\mathrm{of}\;I_{j} \end{array}\right)\times \left(\begin{array}{c} \mathrm{Probability}\;\mathrm{of}\\ \mathrm{transmission}\;\mathrm{per}\\ \mathrm{contact}\;\mathrm{with}\;\mathrm{an}\\ \mathrm{individual}\;\mathrm{in}\;I_{j} \end{array}\right)\times \left(\begin{array}{c} \mathrm{Probability}\\ \mathrm{that}\;a\\ \mathrm{contact}\;\mathrm{is}\\ \mathrm{susceptible} \end{array}\right)\;, \end{array}$$

where $c_{j}$ is the number of contacts an infectious person in compartment $I_{j}$ has per day. The fraction of the contacts with the non-mask- or mask-wearing susceptible is $P_{S}=c_{S}/C_{tot}$. The corresponding force from infection on *S* from an infectious person wearing a mask, $I_{j}^{f}$, is reduced by $r^{i}$ and is defined as $r^{i}\alpha_{j}$.

This force will also be reduced by $r^{s}$ if the susceptible person is wearing a face mask. That is, the force of infection on $S^{f}$ from $I_{j}$ is $r^{s}\alpha_{j}^{f}=c_{j}\left(r^{s}\beta_{j}\right)P_{S}^{f}$, where $P_{S}^{f}=c_{S}^{f}/C_{tot}$. The corresponding force-from-infection from an infectious person wearing a mask, $I_{j}^{f}$, is $r^{i}r^{s}\alpha_{j}$.

The algebraic expression of the all the forces from the infectious are $\alpha_{1}=c_{1}\beta_{1}P_{S},\;\;\;\alpha_{a}=c_{a}\beta_{a}P_{S},\;\;\;\alpha_{m}=c_{m}\beta_{m}P_{S},\;\;\;\alpha_{s}=c_{s}\beta_{s}P_{S},\;\;\;\alpha_{c}=c_{c}\beta_{c}P_{S},\;\;\;\alpha_{1}^{f}=c_{1}\beta_{1}P_{S}^{f},\;\;\;\alpha_{a}^{f}=c_{a}\beta_{a}P_{S}^{f},\;\;\;\alpha_{m}^{f}=c_{m}\beta_{m}P_{S}^{f},\;\;\;\alpha_{s}^{f}=c_{s}\beta_{s}P_{S}^{f},\;\;\;\;\mathrm{and}\;\;\;\;\alpha_{c}^{f}=c_{c}\beta_{c}P_{S}^{f}\;.$ Here, $P_{S}=c_{S}S/C_{tot}$ and $P_{S}^{f}=c_{S}S^{f}/C_{tot}$ are the fractions of the contacts with the non-mask- and mask-wearing susceptible populations. Note that $\alpha_{0}=\alpha_{0}^{f}=0$ since people in $I_{0}$ are not infectious, $\beta_{0}=0$.

#### 2.1.3. Contact Rates

We assume a well-mixed population, and the number of contacts per day that infected individuals have depends on their disease progression state. We assume that all the individuals who are not showing symptoms have the same contact rates, that is, $c_{S}=c_{0}=c_{1}=c_{a}=c_{R}$. We assume that the mildly symptomatic reduce their contacts by a third of the asymptomatic contact rate (Table 1). We distinguish between $I_{s}$ and $I_{c}$ based on their mortality and assume that they have the same contact rates that depend on the household size, $c_{s}=c_{c}$.

We consider each contact as an independent event and do not consider repetitive contacts between individuals. The probability of interaction with a susceptible individual denoted $P_{S}$, is determined by the effective contacts between infected individuals (Figure 1). We note that $P_{S}\neq P_{S}^{f}$ since the probability of running into susceptible non-face mask wearers *S* is different from the face mask wearers $S^{f}$. This probability increases rapidly for a population with a limited number of mask wearers. One comparative advantage to using the force generated from the infectious is the probability of running into one of the stratified susceptible persons is one at the disease-free equilibrium, which corresponds to patient 0 [27].

### 2.2. Model Parameters

The progression rates from compartment *j* to *k* are defined in terms of the mean duration that a person spends within compartment *j* ($\tau_{j}$) and the probability ($P_{jk}$) of progression. We assume that the time, $\tau_{j}$, that a person spends in a compartment is exponentially distributed. This assumption results in a constant transition rate between compartment *j* to compartment *k* of $\gamma_{jk}=P_{jk}/\tau_{j}$ [27]. That is, $\gamma_{01}=1/\tau_{0},\;\;\;\gamma_{1a}=P_{1a}/\tau_{1},\;\;\;\gamma_{1m}=P_{1m}/\tau_{1},\;\;\;\gamma_{ar}=1/\tau_{a},\;\;\;\gamma_{mr}=P_{mr}/\tau_{m},\;\;\;\gamma_{ms}=P_{ms}/\tau_{m},\gamma_{md}=P_{md}/\tau_{m},\;\;\;\;\;\;\gamma_{sr}=P_{sr}/\tau_{s},\;\;\;\;\;\;\gamma_{sd}=P_{sd}/\tau_{s},\;\;\;\;\;\;\gamma_{cd}=P_{cd}/\tau_{c},\;\;\mathrm{and}\;\;\;\;\gamma_{cr}=P_{cr}/\tau_{c}$. We assume that all progression rates are independent of wearing a facial mask.

**Figure 2:**
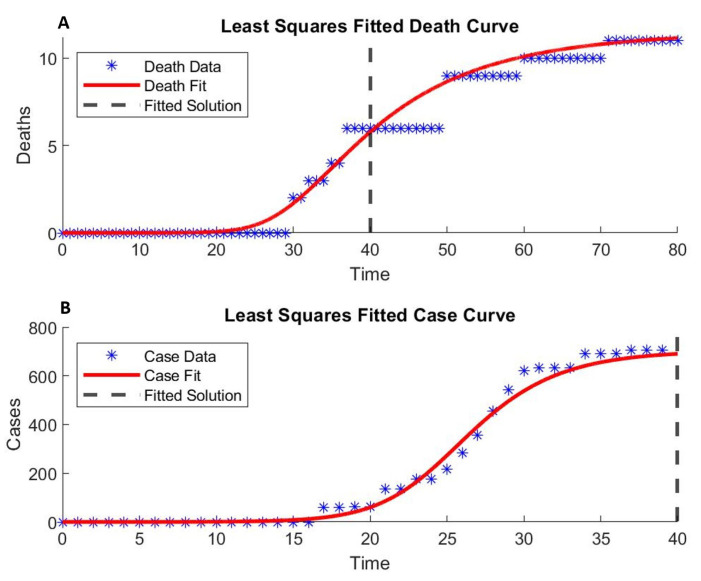
Fitting model parameters with the COVID-19 outbreak data from the Diamond Princess cruise ship [9,26] using our stratified transmission model with a non-linear least square method. (**A**) The cumulative death data from the ship’s departure on 20 January 2020, to disembarkment on 1 March 2020 (40 days), were fitted with $R^{2}=0.983$ and further fitted for five more weeks after disembarkment. (**B**) The positive COVID-19 cases from the ship’s departure to disembarkment (40 days) were fitted with $R^{2}=0.988$. Fitting parameters (Table 1) are estimated with 95% confidence intervals. Details are in Appendix A.

Although we can estimate some of the model parameters from published studies, most studies are for large populations at the country or large city scale. We base our parameters on the most appropriate data we could find and estimated others by fitting the model to the Diamond Princess outbreak data (Figure 2). We first computed the Fisher information matrix to ensure parameter identifiability. The Appendix A include the details of bootstrap re-sampling to estimate parameter sensitivity. Table 1 summarizes the published COVID-19 epidemiology parameters and our estimates for the baseline transmission based on the Diamond Princess outbreak data.

We assume a well-mixed population on board the Diamond Princess cruise ship. The daily contacts for susceptible, pre-, and asymptomatic ($S,\;I_{0},\;I_{1},\;I_{a}$) are identical. Additionally, we assume that people with mild symptoms reduce their daily contact to one-third of the typical number of contacts per day. People with severe symptoms have a daily contact rate of a household with two additional people.

A previous transmission model for the Diamond Princess data estimated the effective contact rate, which is the product between $c_{*}$ and $\beta_{*}$, at 1.41 [28]. Since our model differentiates the presymptomatic, infectious, and asymptomatic infectious, we choose baseline values of $\beta_{1}=0.03$ and consider the infectious after the latent period to have one-third the transmission probability: $\beta_{a}=\beta_{m}=\beta_{s}=\beta_{c}=\beta_{1}/3$. Fitting our model to incorporate transmission differences between presymptomatic and asymptomatic results in insignificant differences.

COVID-19 patients without mechanical ventilation have a mean length of hospital stay ($\tau_{c}$) of 4.8 days [29]. Varying $\tau_{c}$ in our model did not improve the fit. The expected length of stay ($\tau_{s}$) for hospitalized ICU stay was statistically estimated between 15.05 and 19.62 days [30]. We fit the mortality data by stratifying the severe and critical patients into two compartments and assumed no face masks were being worn ($P_{mask}=0$), with 0.37 out of 3700 initially infected.

The basic reproductive number, $R_{0}$, for the Diamond Princess is estimated at values from as high as 14 [28] to values as low as two [7,31]. Our fitted parameters give a $R_{0}=6.31$, which closely resembles the $R_{0}$ observed in Wuhan [1]. We use the complete case data reported (up to 4 weeks from the initial case). Hence, our $R_{0}$ includes mitigation of intervention.

### 2.3. The Reproductive Numbers

The effective reproductive number, $R_{e}$, is the number of new cases infected by a newly infected person during the epidemic. To define $R_{e}$ using the next-generation method [36,37], we first the vector of infection *X* containing all the infected compartments in our model,

$$X={\left[I_{0},I_{1},I_{a},I_{m},I_{s},I_{c},I_{0}^{f},I_{1}^{f},I_{a}^{f},I_{m}^{f},I_{s}^{f},I_{c}^{f}\right]}^{t}\;\;.$$

The differential equations for *X* can be expressed as

$$\frac{dX_{j}}{dt}=F_{j}\left(X\right)-V_{j}\left(X\right)\;.$$

The function F represents the generation of the newly infected in each compartment,

$$F={\left(F_{1},0,0,0,0,0,F_{6},0,0,0,0,0\right)}^{T},$$

where

$$\begin{array}{ccc} F_{1} & = & \alpha_{1}I_{1}+\alpha_{a}I_{a}+\alpha_{m}I_{m}+\alpha_{s}I_{s}+\alpha_{c}\;I_{c}\\ & \; & +r^{j}\left(\alpha_{1}I_{1}^{f}+\alpha_{a}I_{a}^{f}+\alpha_{m}I_{m}^{f}+\alpha_{s}I_{s}^{f}+\alpha_{c}I_{c}^{f}\right)\\ F_{6} & = & r^{s}\left(\alpha_{1}^{f}I_{1}+\alpha_{a}^{f}I_{a}+\alpha_{m}^{f}I_{m}+\alpha_{s}^{f}I_{s}+\alpha_{c}^{f}I_{c}\right)\\ & \; & +r^{j}r^{s}\left(\alpha_{1}^{f}I_{1}^{f}+\alpha_{a}^{f}I_{a}^{f}+\alpha_{m}^{f}I_{m}^{f}+\alpha_{s}^{f}I_{s}^{f}+\alpha_{c}^{f}I_{c}^{f}\right) \end{array}$$

The function V accounts for the transfer of individuals out of each compartment,

$$\begin{array}{cc} V & =\left[\begin{array}{c} \gamma_{1}I_{0}\\ -\gamma_{1}I_{0}+\left(\gamma_{1a}+\gamma_{1m}\right)I_{1}\\ -\gamma_{1a}I_{1}+\gamma_{a}I_{a}\\ -\gamma_{1m}I_{1}+\underset{\gamma_{m}}{\underset{︸}{\left(\gamma_{mc}+\gamma_{ms}+\gamma_{mr}\right)}}I_{m}\\ -\gamma_{ms}I_{m}+\underset{\gamma_{s}}{\underset{︸}{\left(\gamma_{sr}+\gamma_{sd}\right)}}I_{s}\\ -\gamma_{mc}I_{m}+\underset{\gamma_{c}}{\underset{︸}{\left(\gamma_{cr}+\gamma_{cd}\right)}}I_{c}\\ \gamma_{1}I_{0}^{f}\\ -\gamma_{1}I_{0}^{f}+\left(\gamma_{1a}+\gamma_{1m}\right)I_{1}^{f}\\ -\gamma_{1a}I_{1}^{f}+\gamma_{a}I_{a}^{f}\\ -\gamma_{1m}I_{1}^{f}+\underset{\gamma_{m}}{\underset{︸}{\left(\gamma_{mc}+\gamma_{ms}+\gamma_{mr}\right)}}I_{m}^{f}\\ -\gamma_{ms}I_{m}^{f}+\underset{\gamma_{s}}{\underset{︸}{\left(\gamma_{sr}+\gamma_{sd}\right)}}I_{s}^{f}\\ -\gamma_{mc}I_{m}^{f}+\underset{\gamma_{c}}{\underset{︸}{\left(\gamma_{cr}+\gamma_{cd}\right)}}I_{c}^{f} \end{array}\right] \end{array}$$

The basic reproductive number, $R_{0}$, is a special case for $R_{e}$ when everyone in a population is susceptible and not wearing a mask. That is, when $P_{S}=1$, then $R_{0}=R_{e}$. To calculate $R_{0}$, we substitute $P_{S}=1$ and $P_{S}^{f}=0$ into these formulas and then calculate the spectral radius of the next-generation matrix, N [37]. This matrix is defined in terms of the Jacobian matrices as

$$N=J_{F}J_{V}^{-1},\;\mathrm{where}\;J_{F}=\left[\frac{\partial F_{i}}{\partial X_{j}}\right],\;\mathrm{and}\;J_{V}=\left[\frac{\partial V_{i}}{\partial X_{j}}\right].$$

When $P_{S}=1$, the Jacobian matrices are constant since N is linear in $X_{j}$ .

We use the MATLAB symbolic operator to solve for the eigenvalues of N. We then express the transition rates, $\gamma_{*}$, in terms of $\tau_{*}$ and the transition probabilities, $P_{jk}$. Next, we identified products of the transition probabilities and reduced them to a simpler form. That is, because we have assumed that the transition probabilities are independent, we can simplify the equations using the relationship, $P_{ik}=P_{ij}P_{jk}$. For example, $P_{0c}=P_{01}P_{1m}P_{mc}$ is the probability that an infected person in $I_{0}$ will enter $I_{c}$.

After these substitutions, we define $R_{0}$ as the largest eigenvalue of N,

(1) $$R_{0}=\underset{R_{0}\left(I_{1}\right)}{\underset{︸}{P_{01}c_{1}\beta_{1}\tau_{1}}}+\underset{R_{0}\left(I_{a}\right)}{\underset{︸}{P_{0a}c_{a}\beta_{a}\tau_{a}}}+\underset{R_{0}\left(I_{m}\right)}{\underset{︸}{P_{0m}c_{m}\beta_{m}\tau_{m}}}+\underset{R_{0}\left(I_{s}\right)}{\underset{︸}{P_{0s}c_{S}\beta_{s}\tau_{s}}}+\underset{R_{0}\left(I_{c}\right)}{\underset{︸}{P_{0c}c_{c}\beta_{c}\tau_{c}}}\;\;.$$

This basic reproductive number, $R_{0}$, is the expected number of people that a single non-mask-wearing newly infected person will infect in a non-mask-wearing susceptible population. We have decomposed $R_{0}$ into the sum of the expected number of people that a newly infected person will infect while in the infectious compartment, *j*. That is, $R_{0}\left(I_{j}\right)$, is the product of the probability of reaching compartment *j*, $P_{0j}$, times the number of contacts per day for someone in compartment *j*, $c_{j}$, times the probability that a contact with a susceptible person will result in a new infection, $\beta_{j}$, times the number of days spent in the compartment, $\tau_{j}$.

We approximate $R_{e}$ by allowing the probability that a random contact is with a susceptible person to depend on the current state of the population. The forces from infection, $\alpha_{*}$, in F depend on $P_{S}\left(t\right)$ and $P_{S}^{f}\left(t\right)$, and, therefore $R_{e}$ is a nonlinear time-dependent function. Although N is nonlinear in *X*, we assume that the fraction of people in each compartment is slowly varying.

We consider the special case when no one is wearing a mask. For this case, the effective reproductive number is approximated by

(2) $$R_{e}=\underset{R_{e}\left(I_{1}\right)}{\underset{︸}{P_{01}\alpha_{1}\left(t\right)\tau_{1}}}+\underset{R_{e}\left(I_{a}\right)}{\underset{︸}{P_{0a}\alpha_{a}\left(t\right)\tau_{a}}}+\underset{R_{e}\left(I_{m}\right)}{\underset{︸}{P_{0m}\alpha_{m}\left(t\right)\tau_{m}}}+\underset{R_{e}\left(I_{s}\right)}{\underset{︸}{P_{0s}\alpha_{s}\left(t\right)\tau_{s}}}+\underset{R_{e}\left(I_{c}\right)}{\underset{︸}{P_{0c}\alpha_{c}\left(t\right)\tau_{c}}}\;\;.$$

This decomposition for the effective reproductive number, $R_{e}$, illustrates how much each infectious compartment in the model contributes to the spread of the infection during the course of the epidemic. The $R_{e}$ for each compartment can be decomposed into meaningful terms. For example, $R_{e}\left(I_{j}\right)=P_{0j}\alpha_{j}\tau_{j}=P_{0j}c_{j}\beta_{j}P_{S}\tau_{j}$ is the expected number of new infections per day for someone in the infectious compartment $I_{j}$. Here, $P_{0j}$ is the probability of an infected person reaching compartment *j*, $\tau_{j}$ is the average length of time in compartment *j*, and $\alpha_{j}$ is the number of new infections per day from someone in $I_{j}$. Therefore, $\alpha_{j}\tau_{j}$ is the expected number of infections created by someone in $I_{j}$, and $P_{0j}\alpha_{j}\tau_{j}=R_{e}\left(I_{j}\right)$ is the expected number of people that a newly infected person will eventually infect while in $I_{j}$.

If everyone in the population is wearing a face mask, then the masks reduce the effective reproductive number to $R_{e}=r^{i}r^{s}\sum_{j}P_{0j}\alpha_{j}^{f}\left(t\right)\tau_{j}$, where the sum is over all the infected compartments. Similarly, if the susceptible individuals are not wearing masks, but the newly infected person is wearing a mask, then the effective reproductive number is $R_{e}\left(I_{j}\right)=r^{i}\sum_{j}P_{0j}\alpha_{j}\left(t\right)\tau_{j}$.

## 3. Results

### 3.1. Mask Wearing by the Public Flattens the Curve

**Figure 3:**
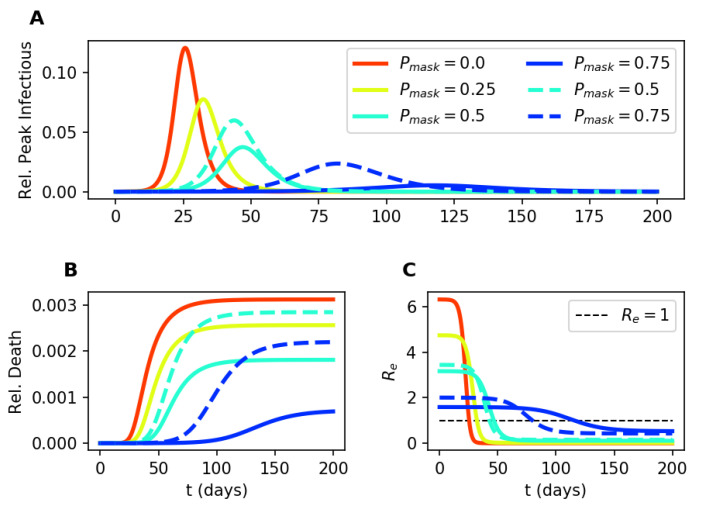
Temporal dynamics of our COVID-19 transmission model for varying proportions of the population wearing the N95 mask ($r^{i}=r^{s}=0.05$) at the start of the simulation. The temporal progression of the peak infectious (**A**), the dead (**B**), and the effective reproductive number $R_{e}$ (**C**). Solid lines correspond to N95 masks used, and dashed lines correspond to cloth masks used. Increasing the fraction of the population wearing the mask ($P_{mask}$) flattens the curve.

To evaluate the contribution of masks in reducing the infectious spread, we measure the population with peak infection, $I_{1}+I_{1}^{f}$, which corresponds to the population tested positive (Figure 3A). As the proportion of mask wearers increases, we observe flattening of the curve where the peak of the infectious population is delayed, and “flattened” [38,39].

When we implement a universal face mask policy across the entire population, i.e., everyone wears the same type of mask, we observe a reduction in both the peak infectious and the dead (Figure 3A,B). As expected, the amplitude of reduction depends on both the fraction of the population wearing masks and the type of masks used. The peak in infections and the total number of deaths are both reduced as more people wear masks. The N95 mask predictions show lower peak infections and fewer deaths than the cloth masks predictions (Figure 3A,B). The model also confirms that $R_{e}$ is reduced as more people wear masks (Figure 3C). We reach *herd immunity* when $R_{e}$ drops below 1 and the infections start to die out. We define the critical inflection time point, $t_{crit}$, as the time when herd immunity ($R_{e}=1$) is achieved. We further analyze how $t_{crit}$ depends on mask wearing in Section 3.4 below.

### 3.2. Face Masks Reduce Deaths

**Figure 4:**
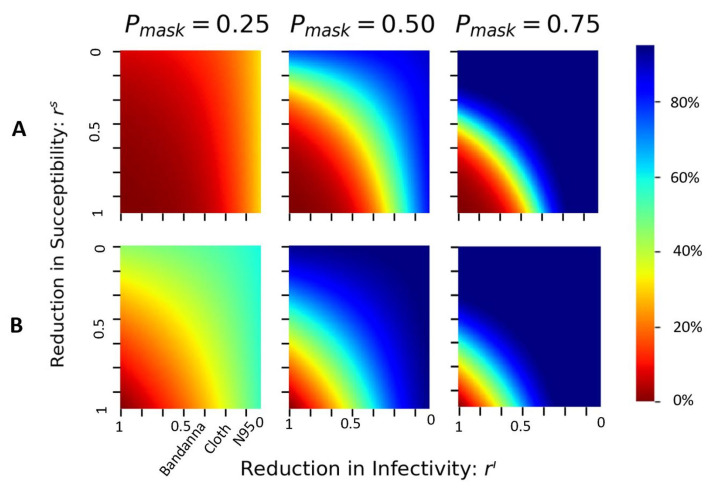
The epidemic slows because of wearing face masks from the embarkment (day 1) of the cruise ship: (**A**) the cumulative infected, and (**B**) the total death at the time of disembarkment (day 40), as a function of reduction in susceptibility $r^{s}$, and reduction in infection: $r^{i}$, for three proportions of mask wearers: $P_{mask}=0.25$ (left), 0.5 (middle), and 0.75 (right).

If wearing face masks starts on the first day (embarkment), the total number of infected cases on day 40 (disembarkment) is reduced by about 30% (Figure 4A) when only 25% of the population wear the N95 masks. This reduction in case number quickly increases to 90% when 50% of the population wears the N95 masks, and the new cases quickly die out when 75% of the population wears a mask. Similarly, the total deaths on day 40 (disembarkment) are reduced by about 50% even when only 25% of the population wears the N95 mask. When 50% of the population wears the N95 mask, the reduction in mortality can be as high as 75% when people use simple face coverings such as bandannas or cloth masks. At 75% compliance, even such simple face coverings could reduce the total death by nearly 100% at the time of disembarkment (Figure 4B).

When the proportion of mask wearers exceeds 50%, the reduction in infection and the reduction in susceptibility offer similar changes in the total cases and deaths, as indicated by the diagonal symmetry in the middle and right columns (Figure 4). However, when a low fraction of the population wears the mask (e.g., $P_{mask}=0.25$, left column in Figure 4), the asymmetrical reduction shows a steeper change along the axis of $r^{i}$. This suggests that when fewer people wear the mask, the infectious spreaders’ masks reduce the epidemic more effectively than if susceptible people wore masks.

When at least 50% of the population wears masks from day 1, we see a significant reduction in both cases and deaths even with simple face coverings (cloth or better); both the case numbers and deaths remain low on day 40, the time of disembarkment. The infection curve is flattened.

### 3.3. A Window of Opportunity to Implement Mask Policy

**Figure 5:**
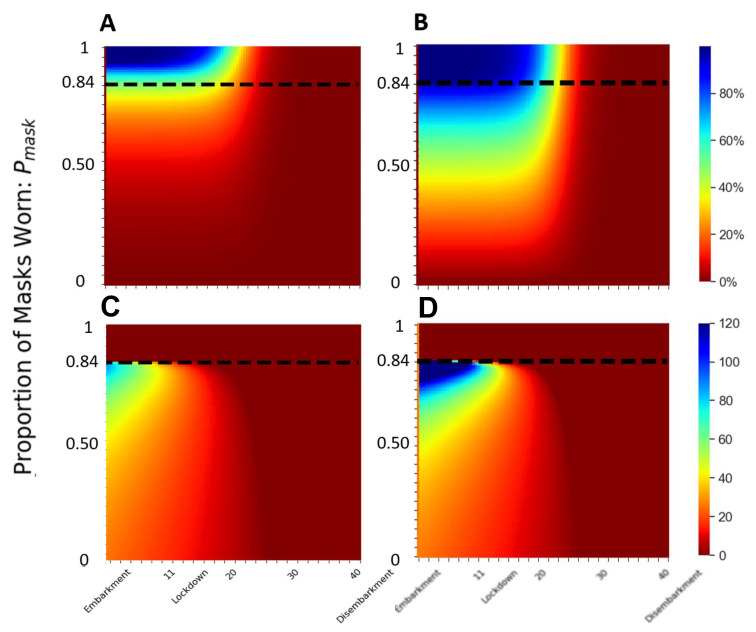
Timing of mask policy implementation. (**A**,**B**) Reduction in the total deaths at the endemic stage when the disease spread is at a steady state (200 days after patient 0), and (**C**,**D**) the critical inflection time $t_{crit}$ when $R_{e}$ drops below 1, as a function of timing of mask implementation and the proportion of mask wearers; (**A**,**C**) for cloth mask ($r^{i}=r^{s}=0.4$), and (**B**,**D**) for N95 mask ($r^{i}=r^{s}=0.05$).

We evaluate the role of timing of mask policy to see how a delayed response will impact the epidemic spread. Following the timeline of the Diamond Princess outbreak, which embarked on 1/25/2020, had the first case of COVID-19 infection identified on 2/3/2020 [28], and started a 14-day quarantine (lockdown) on 2/5/2020 [40], we start the mask intervention on different days between embarkment and disembarkment. The simulations show the reduction in total deaths and cumulative infected at the endemic stage (200 days after the first case, when the outbreak reached a steady state) (Figure 5).

If 75% of the population wears cloth masks from the time of embarkment (day 1), the model predicts that the deaths are reduced by 50%; achieving a similar reduction in mortality with N95 masks needs 50% of the population wearing the mask. When 75% of the population wears N95 masks, the model predicts the deaths are reduced by 66%.

If the mask policy is implemented on the first day of lockdown (day 14), then 84% of the population wearing cloth masks will reduce the total death at endemic by approximately 50%. In comparison, about 52% of N95 mask wearers would achieve a similar reduction (Figure 5A,B). At least 95% of the population needs to wear the mask (cloth or better) to reduce the total deaths by 90%. This finding suggests that the widespread usage of moderate masks during the beginning of the epidemic is more effective than a later application with high-quality masks.

Implementation of mask policy any time between day 1 (embarkment) and day 14 (start of lockdown) does not make a significant change in the reduction of disease spread (Figure 5A). After day 14, achieving the same reduction would require an exponential increase in the proportion of mask wearers for any delay in starting time.

This finding suggests a small window of opportunity to implement a mask policy within 14 days of embarkment for the cruise ship, or within the very narrow window of two days after identifying the first infection, to curb the disease spread effectively. After a week of post-lockdown intervention, wearing masks would have little or no effect on the infectious spread or the mortality on the cruise ship.

### 3.4. At Least 84% Mask Wearers Are Needed to Stop the Epidemic on Diamond Princess

**Figure 6:**
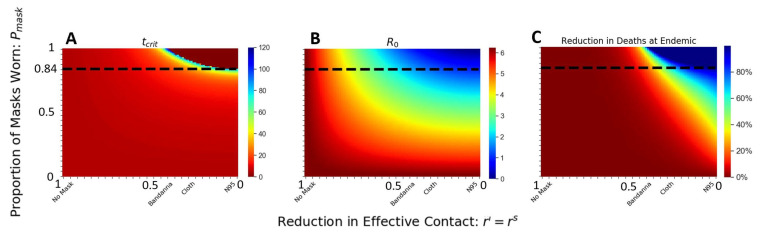
Qualitative phase transition with respect to the fraction of population wearing the mask ($P_{mask}$) and mask efficacy ($r^{i}=r^{s}$): Colors correspond to (**A**) $t_{crit}$, (**B**) $R_{0}$, (**C**) reduction in total deaths, at the endemic stage (200 days after the first case when the epidemic reaches a steady-state). At least 84% of the population needs to wear a mask (cloth or better) to mitigate the epidemic from the start.

We further evaluate the saturation in the infectious spread by identifying the critical inflection time point $t_{crit}$ when $R_{e}=1$, where the disease stops spreading. If no one wears the mask, the critical inflection time point $t_{crit}\approx$ 26 days, about two weeks after the start of the lockdown. Any mask intervention delays this critical inflection time point (Figure 3C). When 84% of the population wears the cloth masks from day 1, the disease spreads for 60 days before $R_{e}$ drops to below 1, and the reduction in the total deaths is about 50%; with N95 masks from day 1, the disease spread takes 4 months until $R_{e}=1$, and the total deaths at endemic is reduced by 100% (Figure 5C,D). If masks were worn within the window of opportunity (from day 1 to day 14), the saturation point occurs when 84% of the population wears the masks (Figure 5). In other words, within the window of opportunity, if at least 84% of the population wears masks (cloth or better), there will be no epidemic. This trend is clear when we examine the basic reproductive number $R_{0}$ (Figure 6B).

This threshold of 84% can be appreciated more clearly in Figure 6: for a fraction of at least 84% wearing the mask and mask quality less than $r^{i}=r^{s}=0.3$ (i.e., any mask better than cloth masks), we see $t_{crit}=0$, $R_{0}<1$, and the reduction in total death is 100%. We have a complete control of the disease: no spread, no epidemic.

This critical threshold point depends on the estimated value of $R_{0}$. Therefore, our qualitative threshold point may apply to other situations with similar homogeneous mixing, although it would not scale linearly with population size. For example, a city or another cruise ship with half of the population density would theoretically cut the mean contacts in half, yet $t_{crit}$ need not scale linearly since the force of infection is nonlinear.

When we have complete compliance with the mask mandate from day 1, $P_{mask}=100\%$, a much wider range of mask types can lead to the complete elimination of epidemic spread. Anything better than $r^{s}=r^{i}=0.45$ will work (Figure 6A–C), which includes bandanna and cloth masks. Bandanna masks have a high variance with an effective reduction between 0.35 and 0.70. The masks made with Cotton Type 3 (cloth) have a narrower range of reduction between 0.20 and 0.40 [41]. Bandanna masks may fall outside the range when cloth masks made with tightly woven fabric are sufficient to stop coronavirus spread, even if the entire population wears them.

For weaker masks, e.g., masks that reduce less than 50% of the air droplets, the disease propagates across the population. Our results suggest a decisive role of the mask in impeding COVID-19 spread, where moderate-quality masks are sufficient to completely stop the epidemic, provided a large proportion of the population wear the masks and are worn within the window opportunity.

## 4. Summary and Discussion

Before April of 2020, the World Health Organization (WHO), the Center for Disease Control and Prevention (CDC), and the European Center for Disease Control (ECDC) recommended hand-washing and social distancing as the main approaches to limit the spread of coronavirus. The guidelines for face masks for the public were enigmatic and changed quickly [42]. The confusing guidelines reduced public trust in public health policies and encouraged much controversy regarding mask usage, from medical risks to political conspiracies. One of the arguments against wearing a mask was that masks would reduce the oxygen for older mask wearers. Solid clinical data have shown that older people’s oxygen saturation does not change before, during, and after wearing non-surgical face masks [43]. We need to better communicate such scientific evidence with the general public to debunk the myths surrounding face masks and good predictive models to encourage better compliance in wearing face masks.

Without sufficiently widespread vaccine coverage, the need for non-pharmaceutical interventions is in high priority [1] to slow down the spread of the COVID-19 pandemic. Countries across the globe have placed strict restrictions on travel and large public gatherings in the form of lockdown interventions [35]. In most cases, travel restriction, school closure, and lockdown interventions offer a significant reduction in the transmission and flatten the curve [39], but come with a hefty economic price. Lockdown may also fail in tightly encapsulated environments with poor ventilation [44]. One convenient, yet controversial, non-pharmaceutical intervention is the mask [7,45,46]. Universal mask usage in combination with conventional lockdown intervention was proposed to offer the greatest non-pharmaceutical intervention for disease-related dynamics [21]. With coronavirus cases still rising, it is important that we settle the debate on masks and that the public uses masks to fight the pandemic.

Masks of different styles and materials have different efficiencies in filtering respiratory particles, including large droplets and smaller aerosols, that carry the coronavirus. The effectiveness of the mask policy is determined by both the mask’s quality and the number of people who appropriately wear fitted masks.

We develop an extended transmission model that treats mask filtration efficiency separately, considering its reduction in susceptibility to incoming particles and infection of outgoing particles. This feature enables us to model the types of masks, the number of people wearing masks, the timing of the mask policy, and who wears the masks. The latter is an important consideration when a low proportion of the population wears the masks, either due to low compliance or a mask shortage. The most commonly used face filtration respiratory mask, the N95 mask, is shown to reduce 95% of virus particles exceeding 0.3 μm. Early at the beginning of the pandemic, many health care institutes reported a shortage of filtration devices for the protection of health care workers [13].

Recent experimental studies have suggested reducing virus spread depends on mask type and how well the masks fit [11]. Masks applied to both receiver and source have been shown to reduce aerosol transmission by up to 96%, while single-fitted medical and cloth masks may only reduce receiver transmission by roughly 50% [12].

We use infection parameters from the coronavirus outbreak in the beginning months of 2020 in the Diamond Princess cruise ship. Respiratory infections are among the common types of outbreaks that occur aboard cruise ships. The outbreak of coronavirus disease in multiple cruise ships globally in 2020 is another notable example.

The Diamond Princess, a tightly encapsulated environment with a relatively homogeneous population, offered a rare set of detailed data of baseline disease transmission and serves as a virtual test-bed for evaluating the role of the mask in mitigating disease transmission. We show that wearing face masks flattens the curve in delaying and reducing the cases and the total deaths from COVID-19. In particular, we identify that a wide supplication of moderate masks homogeneously across all populations at the start of the infection cycle reduces the disease burden more effectively than delayed timing of high-quality masks.

The first 14 days of the itineraries of the cruise ship lay within the window of opportunity to effectively reduce the disease spread by the least amount of wearers and those wearing the lower-quality masks (Figure 5). Furthermore, we demonstrate that the significance of quality of mask choice is most important in the middle of the epidemic as a moderate fraction of people begin to wear masks (Figure 4). Within the window of opportunity, we identify a critical threshold of the percentage of mask wearers at 84%, robust for a wide range of moderate- to high-quality masks (Figure 6). Our results highlight the sufficiency of widespread mask usage from the beginning of the infectious cycle in reducing the infection and the deaths.

## 5. Conclusions

We analyze a homogeneously mixing compartmental SEIAR model with and without masks. Our analytical derivation of the reproductive number (Equation (1)) and the effective reproductive number (Equation (2)) delineates the contribution from each infectious compartment to the spread of the epidemic. This decomposition of $R_{0}$ and $R_{e}$ allows for an analytical understanding of factors influencing the epidemic and efficacy of control policies targeting each infectious subpopulation.

Because we based our parameters on the COVID-19 data from the Diamond Princess data, our simulations can be interpreted as virtual mask experiments on the Diamond Princess. In these virtual experiments, we can vary the fraction of people wearing masks, the types of masks they wear, and the timing of their mask-wearing. We can then compare the spread of the infection and the cases and deaths to those observed on the Diamond Princess.

We apply a uniform mask-wearing policy to the population and evaluate the timing of intervention, quality of the mask, and the fraction of the population wearing the mask. Our results suggest that universal mask is sufficient in reducing the COVID-19 related death and infection (Figure 4) [21]. Specifically, we identify an endemic threshold at 84% of the population wearing the mask, which separating disease-free equilibrium from an endemic state of infection and holds for various mask types (Figure 6). We further evaluate the implementation of the timing of mask intervention and show that an application of moderate-quality mask early achieves similar results to a widespread application of high-quality mask two weeks after initial infection (Figure 5). Our results suggest that in high-risk settings, we need to implement mask policies early, and a critical fraction of the population needs to comply in order to have effective control of the epidemic.

Possible future directions include age-structured population, stratified mask-wearing requirements according to infection status, effect of vaccination, and applying the model to high-risk healthcare settings where healthcare workers and patients should be considered as separate compartments.

## Figures and Tables

**Table 1:** Parameters for the stratified transmission mask model with masks. The boldfaced parameters $\beta_{1},\;P_{1a},\;P_{ms},$ and $P_{sd}$ are fitted from the death data and case data from Diamond Princess using nonlinear least squares (see Appendix B). We defined the mean daily contacts, $c_{1}=61$, as the square root of the capacity of the cruise ship [28]. In the citation column, the E and F indicate if the quantity is estimated or fitted from data.

Parameter	Description	Value & Range	Citation
$c_{S},c_{0},c_{1},c_{a},c_{R}$	Daily contacts for *S*, $I_{0}$, $I_{1}$, $I_{a}$, *R*	61	E
$c_{m}$	Daily contacts for $I_{m}$	20	E
$c_{s},c_{c}$	Daily contacts for $I_{s}$, $I_{c}$	2	E
$\beta_{0}$	Transmission rate for $I_{0}$	0	E
$\beta_{1}$	Transmission rate for $I_{1}$	0.0339; 95% CI: (0.033, 0.034)	F
$\beta_{a},\beta_{m},\beta_{s},\beta_{c}$	Transmission rates for $I_{a}$, $I_{m}$, $I_{s}$, $I_{c}$	$\beta_{1}/3$	E [28]
$\tau_{0}$	Mean time in $I_{0}$	3.69 (days); (3.48, 3.90)	E [32]
$\tau_{1}$	Mean time in $I_{1}$	5.1 (days); (4.5, 5.9)	E [33]
$\tau_{a}$	Mean time in $I_{a}$	$\tau_{1}-\tau_{0}$ (days); (0.60, 2.42)	E [34]
$\tau_{m}$	Mean time in $I_{m}$	5.59 (days); (4.51, 5.86)	E [34]
$\tau_{s}$	Mean time in $I_{s}$	4.8 (days); (2.3, 7.4)	E [29]
$\tau_{c}$	Mean time in $I_{c}$	16 (days); (15.05, 19.62)	E [30]
$P_{10}$	Probability going from $I_{0}$ to $I_{1}$	1	
$P_{1a}$	Probability going from $I_{1}$ to $I_{a}$	0.793; 95% CI: (0.78, 0.81)	F
$P_{ms}$	Probability going from $I_{m}$ to $I_{s}$	0.325; (0, 0.39)	F
$P_{mc}$	Probability going from $I_{m}$ to $I_{c}$	$0.39-P_{ms}$	E [29]
$P_{sd}$	Probability going from $I_{s}$ to *D*	0.0316; 95% CI: (0.026, 0.037)	F
$P_{cd}$	Probability going from $I_{c}$ to *D*	0.117	E [29]
$P_{mr}$	Probability going from $I_{m}$ to *R*	$1-(P_{ms}+P_{mc})$; (0.60, 1)	E
$P_{cr}$	Probability going from $I_{c}$ to *R*	0.883	E [29]
$r^{s}$	Mask reduction in susceptibility	(0, 1)	E
$r^{i}$	Mask reduction in infectiousness	(0, 1)	E
$P_{mask}$	Fraction of population wearing mask	(0, 1)	E
S(0)	Initial susceptible proportion	$0.99999(1-P_{mask})$	E
$I_{0}\left(0\right)$	Initial exposed proportion	$0.0001(1-P_{mask}$	E
$S^{f}\left(0\right)$	Initial susceptible masked proportion	$0.99999P_{mask}$	E
$I_{0}^{f}\left(0\right)$	Initial exposed masked proportion	$0.0001P_{mask}$	E
$R_{0}$	Basic reproductive number	6.31; (5.71,7.23)	E [35]

**Figure A1:**
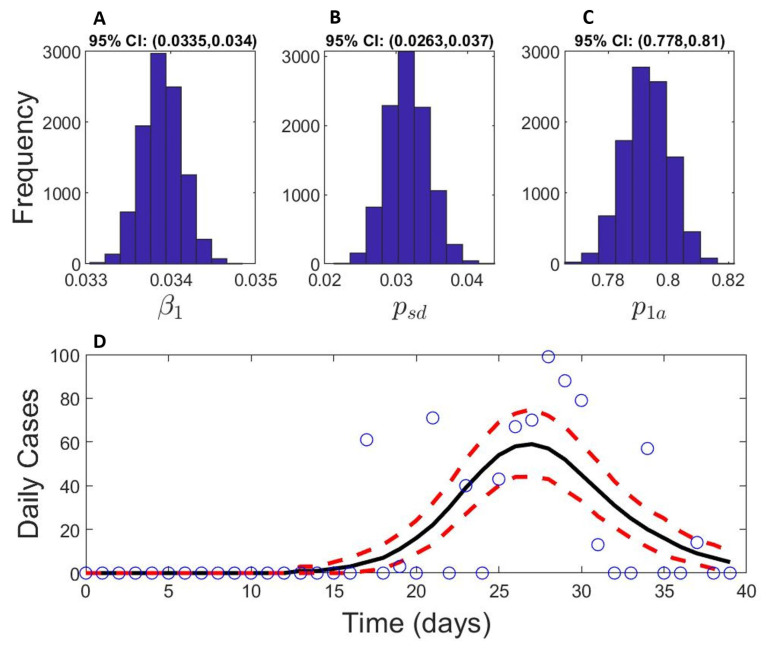
Estimated uncertainty of the fitted model for the first 40 days of COVID-19 cases and deaths in the Diamond Princess: (**A**–**C**) The histograms of the empirical distributions of the fitted parameters $\beta_{1}$ (**A**), $p_{sd}$ (**B**), and $p_{1a}$ (**C**), using 10000 bootstraps realizations for non-linear least squares in MATLAB. (**D**) Estimated daily cases: black line is the best fit; red lines are the 95% confidence bands about the best fit; blue circles are the daily cases data [26]. The range for each parameter corresponds to the 95% confidence intervals (Table 1). The uncertainty is determined on the hypothesis that our model is correct and each parameter assumes values within the range of bounds on the non-linear least squares solver. Probability of severe to deceased: $P_{sd}$ and mild to severe are the most sensitive to our fitted curve in Figure 2.

**Figure A2:**
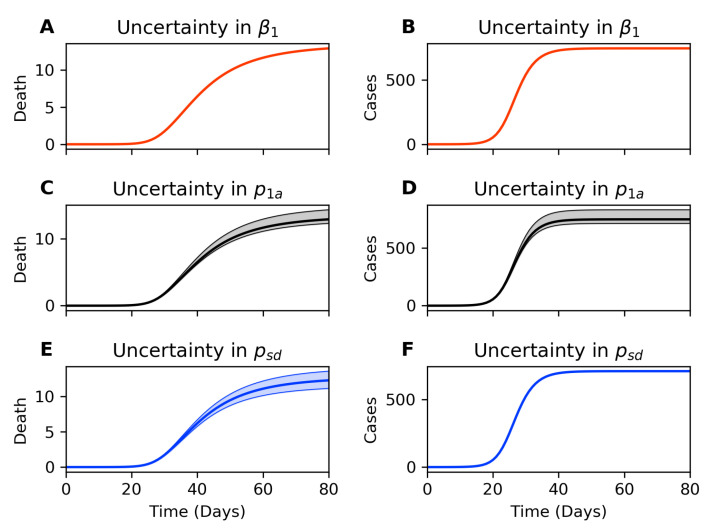
Uncertainty in global sensitivity analysis: 15,000 samples using an acceptance–rejection method. (**A**–**F**): probability density function and cumulative distribution function of the triangle distribution according to the parameters $\beta_{1},\;\beta_{a},P_{1a},\;,P_{ms},\;\mathrm{and}\;P_{sd}$, respectively. The peak of the distribution corresponds to the mean value estimated using nonlinear least squares.

## Appendix A. Global Sensitivity Analysis

We chose to evaluate the global sensitivity using a non-parametric bootstrapping. We generated multiple observations about the fitted solution through sampling. We assume the case count to take on a Poisson distribution with mean at the specific time points $t_{i}$ [47]. We then fit the data using non-linear least squares.

We simulated the model and compared the model predictions with the observed data. The output of the respective parameters (Figure A1) is the empirical distributions of the best-fitted parameters. We fit both the death and case data. We considered the uncertainty in the projection of the death and cases of the best-fitted parameters by computing the trajectories along with the 95% confidence intervals (Figure A2). Note that $P_{1a}$ is the most sensitive parameter within the confidence intervals.

## Appendix B. Local Sensitivity Analysis

We performed local sensitivity analysis (LSA) around our optimal fitted solution (Table 1) to quantify the level of uncertainty in our model. Uncertainties affect the reliability of model predictions and can gauge the scope of the model [48]. The goal of local sensitivity is to evaluate the model’s outputs with respect to key parameters of interest. The most sensitive parameters have the most potent effects for small deviations from an initial value [48].

We perturb our fitted parameters of interest (POIs) around the optimal fitted parameter set and evaluate the change in quantities of interest (QOIs), such as the basic reproductive number ($R_{0}$), deaths at disembarkment, and cumulative infected. For each POI, we determine the sensitivity with respect to each QOI through a sensitivity index (Figure A1C). Comparing parameters of different dimensions is enigmatic unless one quantifies the sensitivity by computing the partials around a local region [48]. The sign of each sensitivity index (Figure A1C) corresponds to the qualitative shift with respect to a magnitude change in the POI. For instance, the sensitivity index of POI $\beta_{m}$ with respect to the QOI: $R_{0}$ is positive, indicating a positive relationship between $\beta_{m}$ and $R_{0}$. The magnitude of the sensitivity index expresses the relative significance of that parameter compared to the others (Table A1). We define a dimensionless sensitivity index by evaluating the response in the QOI (q) relative to a POI (p). Take $\tilde{q}=q(p^{*}$) where $p^{*}$ is the optimal parameter from Table 1. If $p^{*}$ is perturbed locally by $\theta_{p}^{q}$, then the QOI generated with the new parameter set will take the form: $\tilde{q}=\tilde{q}\left(p^{*}+p^{*}\times \theta_{p}^{q}\right)$, where $\theta_{p}^{q}$ is the response generated from a perturbation about $p^{*}:\theta_{p}^{q}=\frac{\partial q}{\partial p}$. We linearize about q and define the sensitivity index as $\frac{p}{q}\times \frac{\partial q}{\partial p}$.

**Table A1:** Parameter sensitivity index for the parameters of interest around the optimal fitted parameters.

Quantity of Interest	$\beta_{1}$	$\beta_{a}$	$P_{1a}$	$P_{ms}$	$P_{sd}$
Infected at Disembarkment	−4.1	−1.5	−0.73	−0.0011	$-2.1\times 10^{-5}$
R${}_{0}$	0.46	0.54	0.33	0	0
Death at Disembarkment	0.86	0.29	−4.1	−2.8	0.43

The optimal fitted parameters of interest are within 10% of its estimated values. The deaths at disembarkment are most sensitive to the probability of transition from infectious and presymptomatic to infectious and asymptomatic ($P_{1a}$). The relative sensitivity index is −4.1, meaning decreasing $P_{1a}$ by 10% would increase the deaths by 4.1%.

The local sensitivity analysis suggests that the mortality is highly sensitive to the fraction of presymptomatic infections that stay asymptomatic infections. The parameters $P_{ms}$ and $P_{mc}$ also significantly contribute to the mortality rate. Our model assumes the identical probability of transmission per contact between mild and asymptomatic symptoms. Hence, we did not choose to vary the value of $\beta_{m}$.

## References

[1] Tang D., Comish P., Kang R. (2020). The hallmarks of COVID-19 disease. PLoS Pathog..

[2] Lerner A.M., Folkers G.K., Fauci A.S. (2020). Preventing the Spread of SARS-CoV-2 with Masks and Other “Low-tech” Interventions. JAMA.

[3] Wang Q., Yu C. (2020). The role of masks and respirator protection against SARS-CoV-2. Infect. Control Hosp. Epidemiol..

[4] Peeples L. (2020). Face masks: What the data say. Nature.

[5] Van Doremalen N., Bushmaker T., Morris D.H., Holbrook M.G., Gamble A., Williamson B.N., Tamin A., Harcourt J.L., Thornburg N.J., Gerber S.I. (2020). Aerosol and surface stability of SARS-CoV-2 as compared with SARS-CoV-1. N. Engl. J. Med..

[6] Wölfel R., Corman V.M., Guggemos W., Seilmaier M., Zange S., Müller M.A., Niemeyer D., Jones T.C., Vollmar P., Rothe C. (2020). Virological assessment of hospitalized patients with COVID-2019. Nature.

[7] Zhang R., Li Y., Zhang A.L., Wang Y., Molina M.J. (2020). Identifying airborne transmission as the dominant route for the spread of COVID-19. Proc. Natl. Acad. Sci. USA.

[8] Scientific Brief, SARS-CoV-2 and Potential Airborne Transmission. https://www.cdc.gov/coronavirus/2019-ncov/science/science-briefs/sars-cov-2-transmission.html.

[9] Global COVID-19 Tracker & Interactive Charts. https://coronavirus.1point3acres.com/en2020.

[10] He X., Lau E.H., Wu P., Deng X., Wang J., Hao X., Lau Y.C., Wong J.Y., Guan Y., Tan X. (2020). Temporal dynamics in viral shedding and transmissibility of COVID-19. Nat. Med..

[11] Brooks J.T., Beezhold D.H., Noti J.D., Coyle J.P., Derk R.C., Blachere F.M., Lindsley W.G. (2021). Maximizing Fit for Cloth and Medical Procedure Masks to Improve Performance and Reduce SARS-CoV-2 Transmission and Exposure, 2021. Morb. Mortal. Wkly. Rep..

[12] Brooks J.T., Butler J.C. (2021). Effectiveness of Mask Wearing to Control Community Spread of SARS-CoV-2. JAMA.

[13] Schumm M.A., Hadaya J.E., Mody N., Myers B.A., Maggard-Gibbons M. (2021). Filtering Facepiece Respirator (N95 Respirator) Reprocessing: A Systematic Review. JAMA.

[14] Hendrix M.J. (2020). Absence of apparent transmission of SARS-CoV-2 from two stylists after exposure at a hair salon with a universal face covering policy—Springfield, Missouri, May 2020. Morb. Mortal. Wkly. Rep..

[15] MacIntyre C.R., Cauchemez S., Dwyer D.E., Seale H., Cheung P., Browne G., Fasher M., Wood J., Gao Z., Booy R. (2009). Face mask use and control of respiratory virus transmission in households. Emerg. Infect. Dis..

[16] Verma S., Dhanak M., Frankenfield J. (2020). Visualizing the effectiveness of face masks in obstructing respiratory jets. Phys. Fluids.

[17] van der Sande M., Teunis P., Sabel R. (2008). Professional and home-made face masks reduce exposure to respiratory infections among the general population. PLoS ONE.

[18] Gandhi M., Rutherford G.W. (2020). Facial masking for COVID-19—Potential for “variolation” as we await a vaccine. N. Engl. J. Med..

[19] Howard J., Huang A., Li Z., Tufekci Z., Zdimal V., van der Westhuizen H., von Delft A., Price A., Fridman L., Tang L. (2021). Face masks against COVID-19: An evidence review. Proc. Natl. Acad. Sci. USA.

[20] Stutt R.O., Retkute R., Bradley M., Gilligan C.A., Colvin J. (2020). A modelling framework to assess the likely effectiveness of facemasks in combination with lock-down in managing the COVID-19 pandemic. Proc. R. Soc. A.

[21] Eikenberry S.E., Mancuso M., Iboi E., Phan T., Eikenberry K., Kuang Y., Kostelich E., Gumel A.B. (2020). To mask or not to mask: Modeling the potential for face mask use by the general public to curtail the COVID-19 pandemic. Infect. Dis. Model..

[22] Kai D., Goldstein G.P., Morgunov A., Nangalia V., Rotkirch A. (2020). Universal masking is urgent in the COVID-19 pandemic: Seir and agent based models, empirical validation, policy recommendations. arXiv.

[23] Tian L., Li X., Qi F., Tang Q.Y., Tang V., Liu J., Cheng X., Li X., Shi Y., Liu H. (2020). Pre-symptomatic Transmission in the Evolution of the COVID-19 Pandemic. arXiv.

[24] IHME (2020). Modeling COVID-19 scenarios for the United States. Nat. Med..

[25] Bai F., Brauer F. (2021). The Effect of Face Mask Use on COVID-19 Models. Epidemiologia.

[26] Ministry of Health Labor and Welfare, Japan. https://www.mhlw.go.jp/content/10200000/Fig2.pdf.

[27] Conrad J.R., Xue L., Dewar J., Hyman J.M. (2016). Modeling the impact of behavior change on the spread of Ebola. Mathematical and Statistical Modeling for Emerging and Re-Emerging Infectious Diseases.

[28] Rocklöv J., Sjödin H., Wilder-Smith A. (2020). COVID-19 outbreak on the Diamond Princess cruise ship: Estimating the epidemic potential and effectiveness of public health countermeasures. J. Travel Med..

[29] Richardson S., Hirsch J.S., Narasimhan M., Crawford J.M., McGinn T., Davidson K.W., Barnaby D.P., Becker L.B., Chelico J.D., Cohen S.L. (2020). Presenting characteristics, comorbidities, and outcomes among 5700 patients hospitalized with COVID-19 in the New York City area. JAMA.

[30] Hazard D., Kaier K., von Cube M., Grodd M., Bugiera L., Lambert J., Wolkewitz M. (2020). Joint analysis of duration of ventilation, length of intensive care, and mortality of COVID-19 patients: A multistate approach. BMC Med. Res. Methodol..

[31] Mizumoto K., Chowell G. (2020). Transmission potential of the novel coronavirus (COVID-19) onboard the diamond Princess Cruises Ship, 2020. Infect. Dis. Model..

[32] Li R., Pei S., Chen B., Song Y., Zhang T., Yang W., Shaman J. (2020). Substantial undocumented infection facilitates the rapid dissemination of novel coronavirus (SARS-CoV-2). Science.

[33] Lauer S.A., Grantz K.H., Bi Q., Jones F.K., Zheng Q., Meredith H.R., Azman A.S., Reich N.G., Lessler J. (2020). The incubation period of coronavirus disease 2019 (COVID-19) from publicly reported confirmed cases: Estimation and application. Ann. Intern. Med..

[34] Bar-On Y.M., Flamholz A., Phillips R., Milo R. (2020). SARS-CoV-2 (COVID-19) by the numbers. eLife.

[35] Tang B., Wang X., Li Q., Bragazzi N.L., Tang S., Xiao Y., Wu J. (2020). Estimation of the transmission risk of the 2019-nCoV and its implication for public health interventions. J. Clin. Med..

[36] Van den Driessche P., Watmough J. (2002). Reproduction numbers and sub-threshold endemic equilibria for compartmental models of disease transmission. Math. Biosci..

[37] Diekmann O., Heesterbeek J.A.P., Metz J.A. (1990). On the definition and the computation of the basic reproduction ratio R_0_ in models for infectious diseases in heterogeneous populations. J. Math. Biol..

[38] OÕDowd K., Nair K.M., Forouzandeh P., Mathew S., Grant J., Moran R., Bartlett J., Bird J., Pillai S.C. (2020). Face Masks and Respirators in the Fight against the COVID-19 Pandemic: A Review of Current Materials, Advances and Future Perspectives. Materials.

[39] Worby C.J., Chang H.H. (2020). Face mask use in the general population and optimal resource allocation during the COVID-19 pandemic. Nat. Commun..

[40] Nakazawa E., Ino H., Akabayashi A. (2020). Chronology of COVID-19 cases on the Diamond Princess cruise ship and ethical considerations: A report from Japan. Disaster Med. Public Health Prep..

[41] Fischer E.P., Fischer M.C., Grass D., Henrion I., Warren W.S., Westman E. (2020). Low-cost measurement of face mask efficacy for filtering expelled droplets during speech. Sci. Adv..

[42] Huo J. Why There Are So Many Different Guidelines For Face Masks For The Public. NPR. https://www.npr.org/sections/goatsandsoda/2020/04/10/829890635/why-there-so-many-different-guidelines-for-face-masks-for-the-public.

[43] Chan N.C., Li K., Hirsh J. (2020). Peripheral Oxygen Saturation in Older Persons Wearing Nonmedical Face Masks in Community Settings. JAMA.

[44] Tokuda Y., Sakihama T., Aoki M., Taniguchi K., Deshpande G.A., Suzuki S., Uda S., Kurokawa K. (2020). COVID-19 outbreak on the Diamond Princess Cruise Ship in February 2020. J. Gen. Fam. Med..

[45] Wang J., Pan L., Tang S., Ji J.S., Shi X. (2020). Mask use during COVID-19: A risk adjusted strategy. Environ. Pollut..

[46] Nicola M., Alsafi Z., Sohrabi C., Kerwan A., Al-Jabir A., Iosifidis C., Agha M., Agha R. (2020). The socio-economic implications of the coronavirus pandemic (COVID-19): A review. Int. J. Surg..

[47] Chowell G. (2017). Fitting dynamic models to epidemic outbreaks with quantified uncertainty: A primer for parameter uncertainty, identifiability, and forecasts. Infect. Dis. Model..

[48] Arriola L., Hyman J.M. (2009). Sensitivity analysis for uncertainty quantification in mathematical models. Mathematical and Statistical Estimation Approaches in Epidemiology.

